# Consensus Rules in Variant Detection from Next-Generation Sequencing Data

**DOI:** 10.1371/journal.pone.0038470

**Published:** 2012-06-08

**Authors:** Peilin Jia, Fei Li, Jufeng Xia, Haiquan Chen, Hongbin Ji, William Pao, Zhongming Zhao

**Affiliations:** 1 Department of Biomedical Informatics, Vanderbilt University School of Medicine, Nashville, Tennessee, United States of America; 2 State Key Laboratory of Cell Biology, Institute of Biochemistry and Cell Biology, Shanghai Institutes for Biological Sciences, Chinese Academy of Sciences, Shanghai, China; 3 Department of Thoracic Surgery, Fudan University Shanghai Cancer Center, Shanghai, China; 4 Department of Oncology, Shanghai Medical College, Shanghai, China; 5 Vanderbilt-Ingram Cancer Center, Vanderbilt University Medical Center, Nashville, Tennessee, United States of America; 6 Department of Medicine/Division of Hematology-Oncology, Vanderbilt University School of Medicine, Nashville, Tennessee, United States of America; 7 Department of Psychiatry, Vanderbilt University School of Medicine, Nashville, Tennessee, United States of America; University of California, Los Angeles, United States of America

## Abstract

A critical step in detecting variants from next-generation sequencing data is *post hoc* filtering of putative variants called or predicted by computational tools. Here, we highlight four critical parameters that could enhance the accuracy of called single nucleotide variants and insertions/deletions: quality and deepness, refinement and improvement of initial mapping, allele/strand balance, and examination of spurious genes. Use of these sequence features appropriately in variant filtering could greatly improve validation rates, thereby saving time and costs in next-generation sequencing projects.

## Introduction

The recent successful applications of next-generation sequencing (NGS) technologies to identify disease-associated variants have revolutionized biomedical and biological research, especially in human disease studies [1], [2]. Rapid advances in NGS technologies, along with the dramatic decrease of cost, have propelled them to become a major approach in research. As of September 2011, more than 40 Mendelian diseases have been analyzed using whole exome sequencing (WES) [3], and more than 10 complex diseases have been studied using whole genome sequencing (WGS) and/or WES, including, but not limited to, renal cancer [4], melanoma [5], hepatocellular carcinoma [6], acute monocytic leukemia [7], and head and neck squamous cell carcinoma [8]. While most of the early NGS studies were conducted by large sequencing centers or prominent research groups [2], [9], the tremendous improvement in technologies during the past two to three years has dramatically reduced cost and hastened the speed of sequencing a genome within a short period of time. Subsequently, NGS technologies are now affordable and accessible to small or moderately sized laboratories and are expected to quickly evolve as a routine experimental technique in a similar fashion as the now common use of microarray.

NGS generates massive amounts of data for genetic variant detection. Thus, currently, a major bottleneck of NGS applications is downstream bioinformatics analysis. This problem is especially challenging for bench scientists. To meet this strong demand, many investigators have been redirected to this new field and are in the early stages of acquiring this technological knowledge, especially pertaining to data analysis. Meanwhile, a great number of computational tools dedicated to almost all aspects of NGS data analyses have been developed during the past few years. However, these tools are complicated and have project-specific features. Firsthand experience in using these tools is important, especially because investigators need to be able to readily identify true variants for validation from the typically millions of variants called by NGS computational tools. In this paper, we discuss four major parameters that affect variant calling and the validation process, aiming to provide some general guidelines to the NGS community, especially for those new to NGS applications.

**Figure 1:**
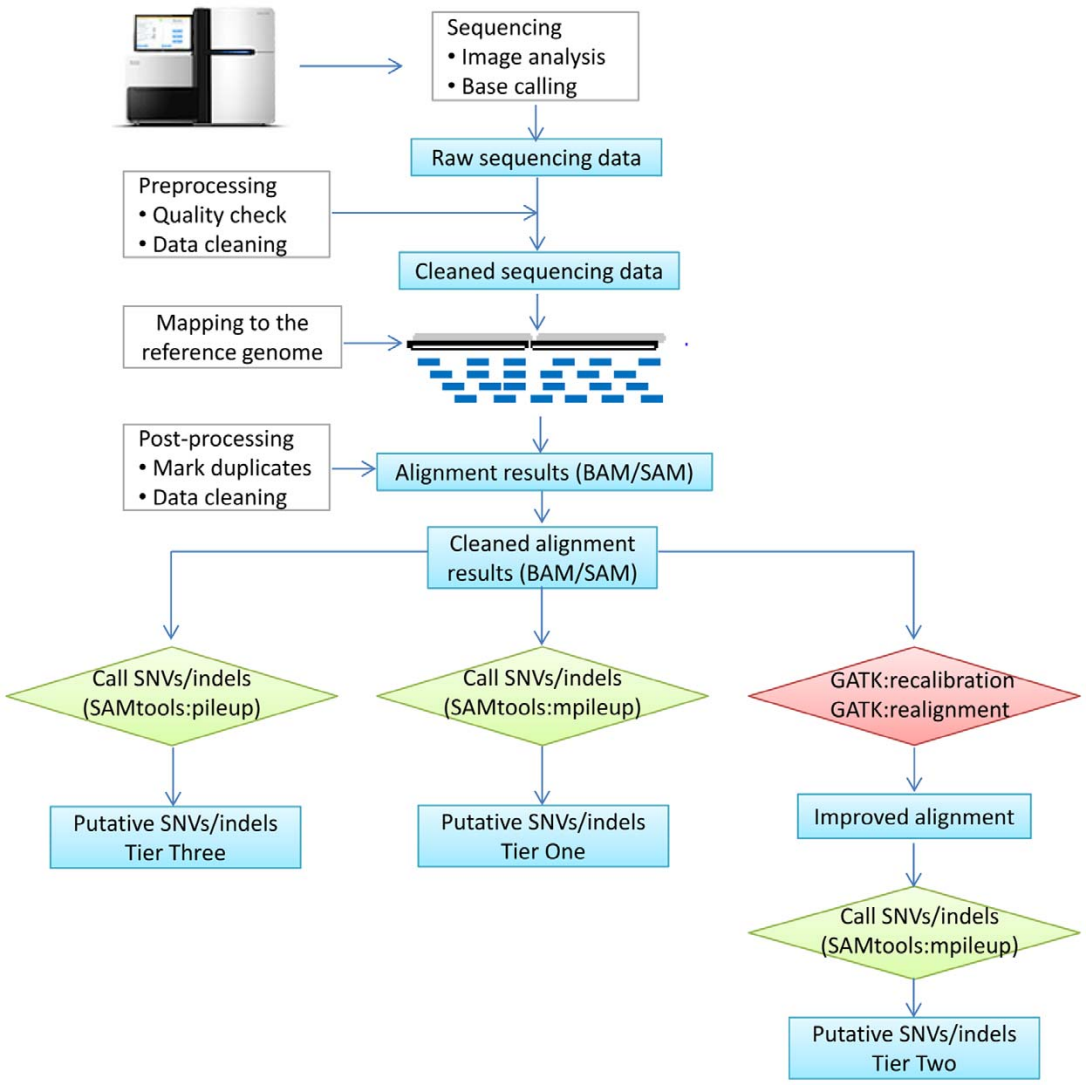
Pipelines for calling SNVs and indels. SNVs and indels are called by three options based on SAMtools (pileup or mpileup) and GATK recalibration. Accordingly, three tiers of SNVs and indels are used for comparison. SNVs: single nucleotide variants. Indels: insertions and deletions.

Among the various types of mutations that cause diseases, single nucleotide variants (SNVs) and small insertions and deletions (indels) are the most abundant. The detection of these variants is critical in both WGS and WES studies. In the NGS data analysis pipeline, SNV/indel calling is performed after mapping reads to a reference genome, typically generating an initial set of SNVs/indels. Based on several recently sequenced individual genomes [10], [11], [12], a pattern has been recognized that, in general, approximately 3–4 million SNVs are expected to be found in a human genome by WGS when compared to the reference genome [13], and ∼20,000 SNVs are to be found in a human exome by WES [14]. Some of these SNVs might, however, be false positives. An open question is how to identify a set of SNVs with high enough quality for follow up validation or further analysis. In an early study by Ley et al. [15], the authors generated an initial calling list of SNVs (∼3.8 million SNVs) using the software Maq [16] and then selected a subset of mutations from the list for experimental validation. After they used their experimental data as a training dataset for the Decision Tree C4.5 algorithm, the authors successfully learned a set of critical rules (e.g., based on read counts, base quality and SNP quality scores), which were then used to predict a small yet well supported set of ∼2.6 million SNVs with high accuracy [15]. In another study, Wei et al. [5] called SNVs using the software bam2mpg [17] and developed a ratio score to evaluate the quality of the initially called genotypes. Based on their experimental data, the authors estimated a threshold of this ratio score and used it to filter their initial list of SNVs.

**Table 1:** Comparison of validation of 159 SNVs and 22 indels by different parameter setting in variant calling.

Dataset	Condition	TP	FP	TN	FN	Validate rate	Recall	Accuracy	
*SNVs (159)*									
Tier One	Initial calling	65	−	94	−	65/159 = 40.88%			
Tier One	QUAL≥28, DP≥5	50	9	85	15		50/65 = 76.92%	50/59 = 84.75%	
Tier Two	No filtering	65	80	14	0		65/65 = 100%	65/145 = 44.83%	
Tier Two	QUAL≥23, DP≥5or QUAL≥25, DP≥3	59	10	70	6		59/65 = 90.77%	59/69 = 85.51%	
*Indels (22)*									
Tier One	Initial calling	12	−	10	−	12/22 = 54.54%			
Tier One	QUAL≥17, DP≥3	12	1	9	0		12/12 = 100%	12/13 = 92.31%	
Tier Two	No filtering	9	10	0	3		9/12 = 75%	9/19 = 47.37%	
Tier Two	QUAL≥21, DP≥3	9	0	10	3		9/12 = 75%	9/9 = 100%	

QUAL: quality score for SNVs and indels. DP: read depth. Definition of Tiers One and Two is provided in text and Figure 1. TP: true positive; FP: false positive; FN: false negative; TN: true negative;
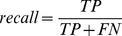
; 
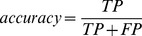
.

These two studies, as well as several large-scale sequencing projects [9], [14], have shown that post-filtering of SNVs is essential to identify variants that are more likely to be true while effectively filtering false positives. Although not formulated, a few consistent filtering rules have been recognized by the community, including base quality, mapping quality, and coverage of supporting reads. Additionally, there are several other factors that may affect the accuracy of SNV/indel calling, such as sequence complexity and the fitness of the algorithms used in a specific case. In this study, we discuss four parameters that affect SNV and small indel calling, which are critical in NGS applications. These parameters are (1) variant quality and coverage, (2) refinement and improvement of initial mapping, (3) allele/strand balance, and (4) examination of spurious genes. Although some of them have been discussed in previous studies in various forms [14], [18], [19], here, we systematically examined and demonstrated these rules using our own experimental data, so that they may be generally applied to different NGS data analyses.

## Results and Discussion

### Analysis Pipeline for SNV/Indel Detection

A straightforward, yet concise, pipeline to detect SNVs and indels includes (1) sequencing, (2) pre-processing (e.g., quality check, data cleaning if necessary), (3) mapping reads to the reference genome using tools like the Burrows-Wheeler Alignment tool (BWA) [20], Bowtie [21], or others, (4) post-processing of the alignment results (e.g., marking duplicates, sorting, and indexing), (5) calling SNVs/indels using tools such as SAMtools [19], [22] and/or the Unified Genotyper implemented in the Genome Analysis Toolkit (GATK) [14], and (6) filtering (Figure 1). Advantages of this workflow include that it is straightforward, uses robust technologies (applicable to both WGS and WES), is fast to execute especially for WGS data, and leads to an acceptable false positive rate. In our implementation, we applied BWA (version 0.5.9) to map reads using all default parameters. The Picard tool (version 1.52) was used to mark duplicates, and the SAMtools:*mpileup* function (version 0.1.13) was used to call SNVs/indels. In the filtering step, we used “*perl vcfutils.pl varFilter*” with a “*-d 3 -D 10000*” option and other parameters by default and denoted the results using this pipeline as the tier one variant set (see the Materials and Methods section for details). We did not apply any further filtering rules to the tier one variants, as our intention was to search for parameters and rules that are effective for improving the validation rate. For this purpose, we required an unbiased set of variants for validation.

Alternatively, the GATK program can be incorporated before variant calling. While GATK has a comprehensive list of functions for almost all the NGS analyses, here, we focused on an enhancement of our pipeline using GATK (Figure 1). Specifically, we proposed the incorporation of GATK in the step after read mapping and before SNV/indel calling by SAMtools:*mpileup*. Among all the complicated tools implemented in GATK, we found that two functions, the recalibration of mapping scores and local realignment around indels, were particularly useful to improve initial mapping results before SNV calling. After the integration of these two steps, a new set of mapping results in BAM/SAM format [22] were generated and analyzed using the SAMtools:*mpileup* function for calling SNVs/indels, followed by *varFilter* for initial filtering. We denoted the results as the tier two variant set.

Of note, the function to call SNVs and indels in SAMtools previously was *pileup*, which became obsolete since version 0.1.10 and was replaced by *mpileup*. Thus, most studies published in early 2011 or before used SAMtools:*pileup*. To compare performance between these programs, we also implemented SAMtools:*pileup* (version 0.1.13, *pileup* was still available in this version); results were denoted as the tier three variant set.

### Validation Dataset

We used real data generated from NGS of 18 tumor-normal pairs using an Illumina HiSeq 2000 (Chen, Pao, Zhao and Ji, unpublished data). Starting with the tier one variant set, we selected a total of 181 mutations based on potential functional importance, including both SNVs and indels called in the 18 cancer samples. The tier one set was generated based on the most straightforward analysis pipeline, i.e., no post-improvement on the alignment results or any filter rules on the initial results; thus, it included almost all putative variants directly obtained from the pipeline output. The false positive rate was expected to be high. Among these 181 mutations, 159 were SNVs and 22 were indels (Table 1). Primers were designed for each of these variant sites, and traditional Sanger sequencing was used to sequence the corresponding PCR products. Sixty-five of the SNVs were validated as true variants, with a validation rate of 40.88%. Similarly, 12 indels were validated, and the validation rate was 54.54%. Overall, the validation rate was low for SNVs/indels detected without applying filtering.

To perform a systematic evaluation of the variants by three tiers, we used the following indicators to distinguish different cases of prediction and validation data.

- True Positive (TP): variants predicted and validated.
- False Positive (FP): variants predicted but failed in validation.
- False Negative (FN): variants not predicted but validated.
- True Negative (TN): variants not predicted and not validated.

Three parameters were introduced in our evaluation: 
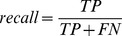
, 
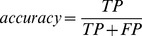
, and an *F* score, 
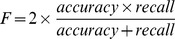
. Due to the limitation of the dataset, we could not obtain an accurate computation for the area under the receiver curve (AUC), a widely used term in data mining. Rather, we incorporated the *F* score to assist with the selection of parameters and create a balance between accuracy and recall. Based on our evaluation, we summarized the following four points that are critical for improving variant call.

### Point 1: Both Quality and Read Depth Matter

In early applications using SAMtools to call SNVs/indels, cutoff values of 20 for SNV quality (hereafter denoted as QUAL) and 50 for indels were suggested [23]. In the recent versions of SAMtools, *mpileup* has replaced *pileup* with a newly introduced concept of Base Alignment Quality (BAQ) [19]. We were unable to find an explicit recommendation for the cutoff values that are appropriate for filtering putative SNVs/indels. To find the appropriate cutoff values for *mpileup*, specifically for SNVs, we systematically compared the QUAL values for the variants called by SAMtools:*mpileup* (tier one set) and SAMtools:*pileup* (tier three set) for each of the 18 cancer samples. The Pearson correlation coefficients were very high and in a small range (0.9872 to 0.9946) among the 18 samples, although SAMtools:*pileup* QUAL scores were slightly higher than SAMtools:*mpileup* scores in >80% cases. Results of one sample are provided in Figure S1. This comparison indicates that the QUAL scores of variants called by *mpileup* and *pileup* are quite similar. Accordingly, we suggest a cutoff QUAL value ∼20 can be generally applied to variants called by SAMtools:*mpileup*.

**Figure 2:**
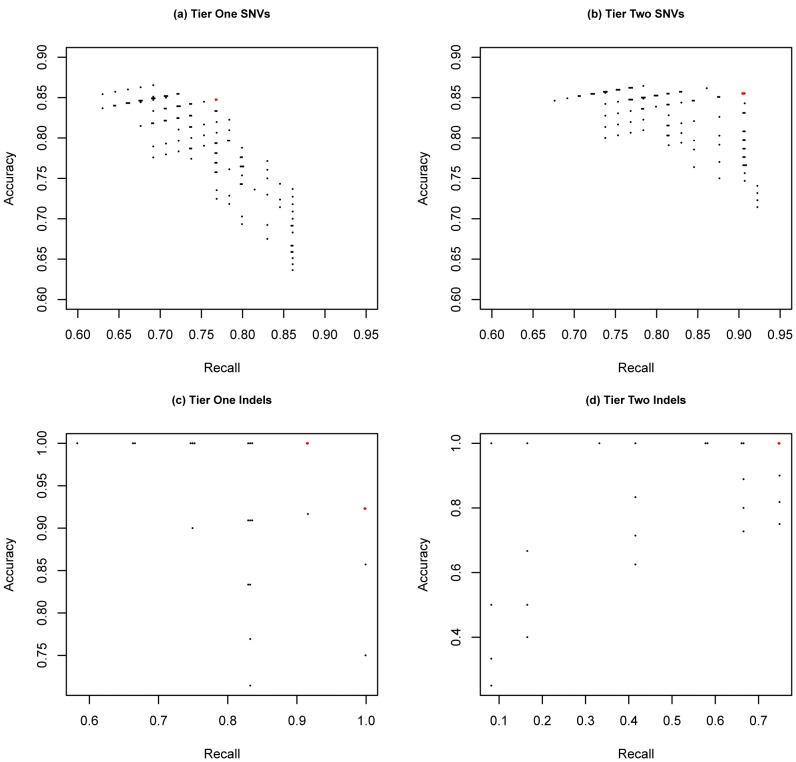
Distribution of accuracy versus recall by different combinations of quality score (QUAL) and read depth (DP) values in two sets (tiers 1 and 2) of SNVs and indels. (a) Tier One SNVs. (b) Tier Two SNVs. (c) Tier One Indels. (d) Tier Two Indels. For each variant set (panel), each node represents a combination of cutoff values for QUAL and DP. Specifically, the QUAL cutoff was selected by an integer value in the range of 15 to 35 with an increment of 1 each time, and the DP cutoff by an integer value in the range of 3 to 15 with an increment of 1 each time. Then, we evaluated the accuracy, recall, and *F* score (see text) for each cutoff combination. Note that many nodes are overlapped on the panel and shown by jitter (i.e., points at the same locations are slightly shifted for visibility). The combination of values that could generate the highest *F* score was selected (shown in red points).

**Figure 3:**
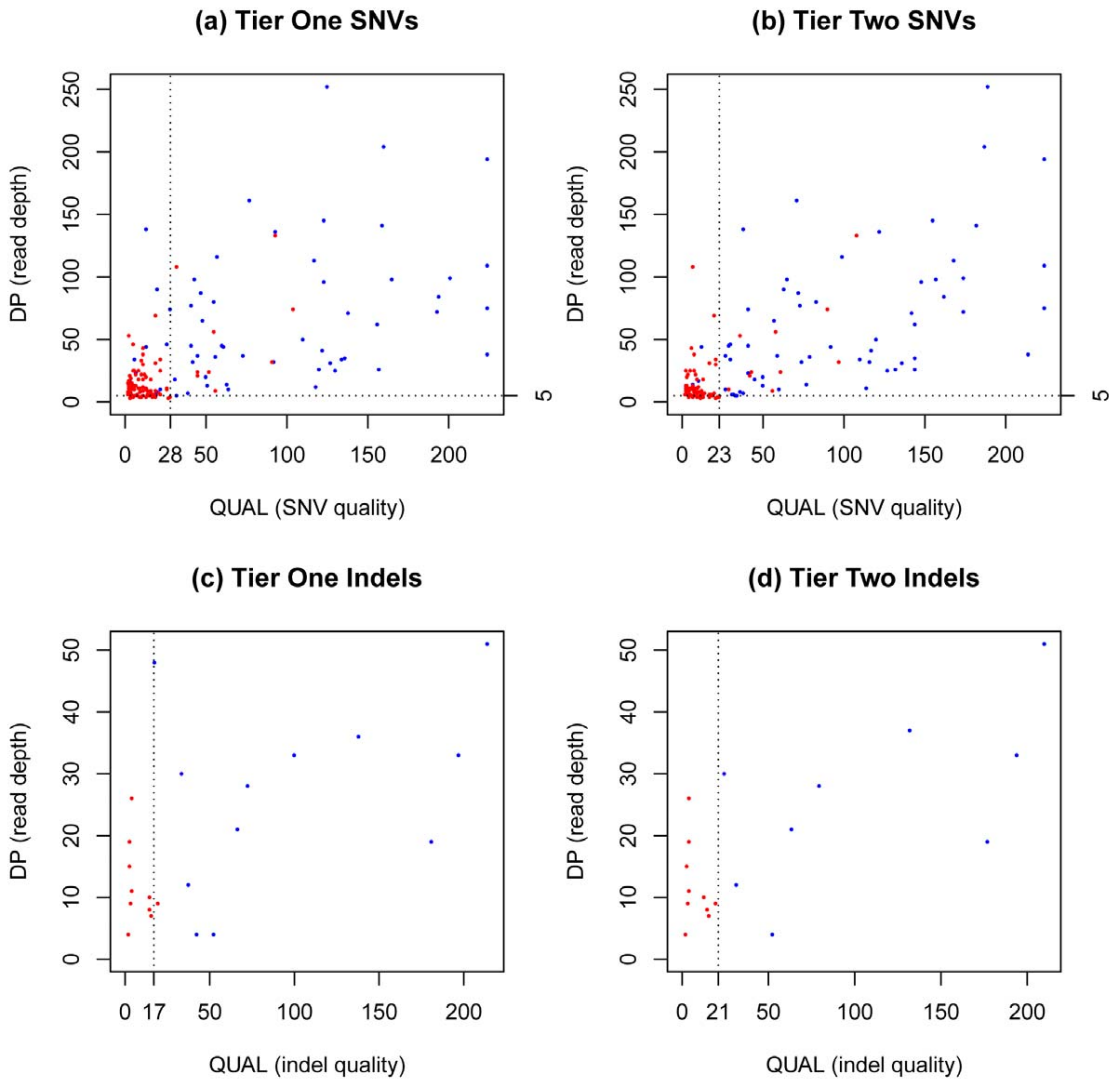
Distribution of read depth (DP) versus SNV quality score (QUAL) for the SNVs or indels selected for validation. (a) Tier One SNVs (159 SNVs), (b) Tier Two SNVs (145 SNVs), (c) Tier One Indels (22 indels), and (d) Tier Two Indels (19 indels). Variants in blue denote successful validation, and variants in red denote failure in validation. In each panel, the vertical dash line indicates the cutoff value for QUAL, and the horizontal dash line indicates cutoff value for DP (see Point 2 in the main text and Table 1).

To further explore which cutoff values are most appropriate, we compared the performance of variant calling through our validation dataset. Specifically, initial variants were separated into different groups through possible combination values of QUAL and read depth (DP). We limited QUAL values in the range between 15 and 35 with a step-wise increase of 1 and DP in the range between 3 and 15 with step-wise increase by 1 as well. The recall, accuracy, and *F* score for each of the possible combinations of QUAL and DP were computed and compared, with the goal of finding the combination of QUAL and DP scores that could generate the highest *F* score. For tier one variants, we found that with QUAL ≥28 and DP≥5, the highest *F* score (0.8065) could be achieved and a total of 50 of the 65 validated SNVs could be recruited (recall  = 76.92%), with an accuracy rate of 84.75% (Figures 2a and 3a and Table 1). For tier one indels, using QUAL ≥17 and DP≥3, all 12 validated mutations could be recalled (100%), with an accuracy rate of 92.31% (Table 1, Figures 2c and 3c). Although the *F* score may not be the most appropriate for identifying QUAL and DP cutoff values, its notion of making a trade-off between accuracy and recall is reasonable. We expect to further improve this evaluation approach for identifying cutoff values. For example, another combination for tier one indels, QUAL ≥21 and DP≥3, could generate a higher accuracy (100%) with a slight decrease in the recall rate: only 11 indels could be recalled (11/12 = 91.67%) (Figure 2c). Therefore, if high accuracy is a high priority and resources allow only a limited number of variants to be validated, the options “QUAL ≥21 and DP≥3” is preferred. On the other hand, if the goal is to search for and validate as many possible variants with abundant resources available, “QUAL ≥17 and DP≥3” could be adopted.

### Point 2: Realignment and Recalibration Improve Variant Calling

When using the validation data to compare variant calling in the tier one and two sets, we had better performance measured by both recall and accuracy, especially for SNVs. With QUAL ≥23 and DP≥5, or QUAL ≥25 and DP≥3, we could recruit 59 tier two SNVs (recall  = 90.77%) with an accuracy of 85.51% (Figures 2b and 3b). This result was compared to the highest recall rate (76.92%) and accuracy rate (84.75%) using tier one SNVs. For indels, using QUAL ≥21 and DP≥3, only 9 of the 12 validated indels were recruited (Figures 2d and 3d), which is slightly lower than the tier one data where all 12 validated indels could be recruited and the accuracy was 100%.

For both tier one and tier two sets, the cutoff values we proposed here might be variant set-specific and may vary according to specific conditions; thus, there is no need to follow the exact values. Rather, our values here demonstrate, based on our data and experience, that a cutoff around 20–25 for SNV quality and read depth ≥5, or higher, if data allows, would lead to a high validation rate when using the pipeline for the tier two variant set.

**Figure 4:**
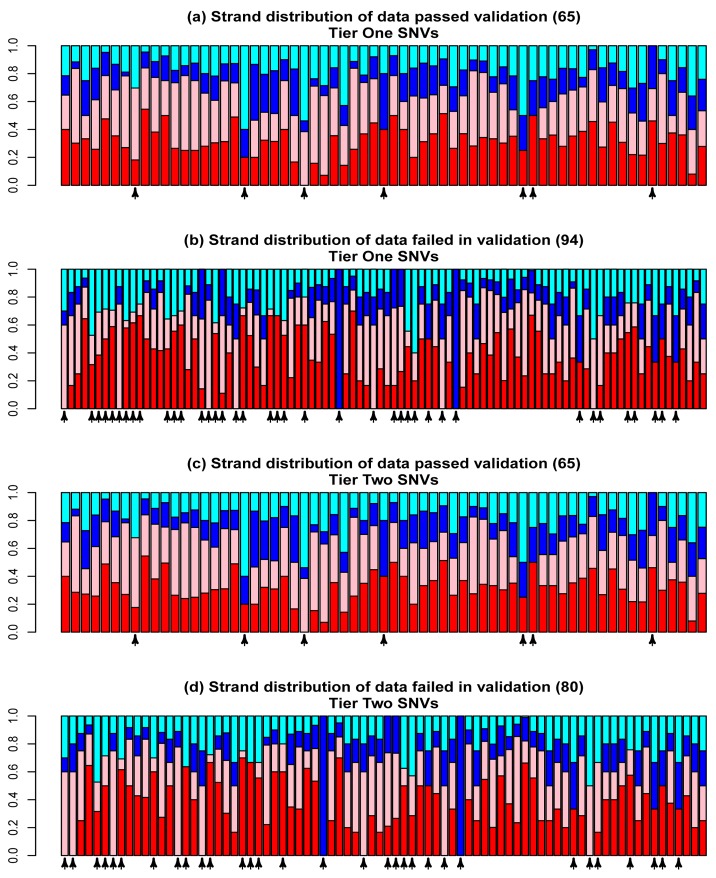
Allele and strand bias for SNVs. This figure shows read distribution of called variants to reference or alternative (i.e., non-reference) alleles in forward or reverse strand. (a) Tier One SNVs passed validation. (b) Tier One SNVs failed in validation. (c) Tier Two SNVs passed validation. (d) Tier Two SNVs failed in validation. Red: reference base forward; pink: reference base reverse; blue: alternative base forward; and cyan: alternative base reverse. The arrows under the x-axis indicate the variants lacked supporting reads for one or more of the four allele/strand cases.

### Point 3: Allele and Strand Bias

**Figure 5:**
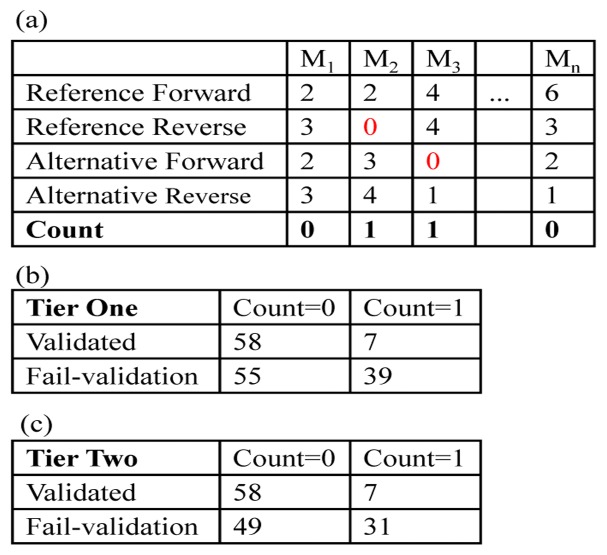
An illustration of Fisher’s exact test for allele and strand balance. On the top panel (a), the table shows how we summarized the counts for each mutation site (shown in each column and denoted by M) in each of the four cases: reference forward, reference reverse, alternative forward, and alternative reverse. A variant is indicated by 1 if it does not have a supporting read in one or more cases; otherwise, it is indicated by 0. The contingency tables for the Tier One dataset and Tier Two dataset were constructed as shown in (b) and (c), respectively.

Other factors that have been previously mentioned [14], [24] include a required supporting read number regarding the reference or alternative alleles in the forward and reverse strands, respectively. We systematically examined the allele and strand distribution of the validation data in the tier one and two variant sets (Figure 4). For each variant site, four numbers of “high-quality” reads were obtained respectively for (1) reference allele forward (RF), (2) reference allele reverse (RR), (3) alternate allele forward (AF), and (4) alternative allele reverse (AR). Here, “high-quality reads” indicate those that were literally used in SNV/indel calling by SAMtools:*mpileup* function and were reported as the “DP4” item in the resultant files in VCF format [25]. As shown in Figure 4, we indeed observed a strong difference between the variants passing validation and those that failed in validation. To describe this quantitatively, in each scenario, we counted the number of variants with at least one supporting read for all four allele/strand combinations and those with no supporting reads in any of the four combinations (Figure 5a), and then constructed 2×2 contingency tables (Figure 5b and 5c). Fisher’s exact test showed that there was a significant difference between the read distribution and validation status (*P*  = 1.76×10^−5^ for tier one variant set and *P*  = 1.27×10^−4^ for tier two variant set). However, when we attempted to use this rule to perform prediction, i.e., requiring at least one supporting read for each of the 4 base/strand combinations, we did not find that recall or accuracy improved substantially. Thus, no filtering rules based on the allele/strand balance were explicitly applied in our analysis. Even so, this lack of improvement might occur in our data specifically. Overall, we suggest that researchers check the allele/strand bias in their own projects.

### Point 4: Manual Check of Spurious Genes

Previous studies have noticed the phenomenon of spurious genes (genes with spurious mutations found in many samples or different projects) caused by similar regions in the genome [18]. These genes, e.g., *CDC27*, *CTBP2*, and *OR4C3*, have been frequently predicted, in different projects, to have mutations, but these findings were finally proved to be artifacts [1], [26], [27]. The details of spurious genes in NGS data have yet to be explored. A possible scenario is described below. Suppose there are two regions A and B in the genome. Region A is included in the current version of the reference genome, but region B is not due to the incompleteness of our knowledge. Using currently available mapping tools, the reads that are initially generated by region B will hardly be confidently mapped to any region in the genome and will be discarded. However, if the similarity between the two regions is very high and the read is short, it would be possible that the reads that are originally generated by region B will be assigned to region A with mismatches, and these “mismatches” could subsequently be reported as putative SNVs/indels in region A. Ju et al. [28] proposed “super” genes to classify this type of gene, which was found to have a high density of detected SNVs in their genomic regions. We observed that *CDC27* was frequently predicted to have mutations in several of our in-house exome sequencing projects with different phenotypes (data not shown). It is important to distinguish such genes, especially when sequencing a number of cancer samples, because in such conditions, investigators would be particularly interested in searching for genes with a high mutation frequency in multiple samples. Without warning, it is likely that investigators will identify top candidate genes with a high frequency of spurious mutations. Should this occur, such artifacts could waste a lot of resources in validation work, or even lead to false discovery reporting in the literature.

**Figure 6:**
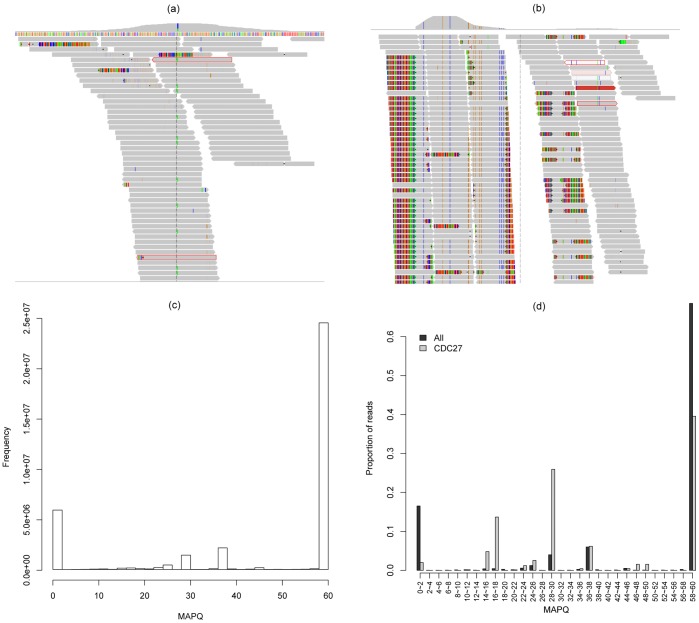
A visual examination of a spurious gene (*CDC27*). The top panels show visualization of read alignment in good (a) and bad (b) conditions using the software IGV [29]. The top part of each figure shows the coverage. Each grey bar represents one read, with the color grey indicating it is matched well with the reference and other colors indicating mismatches. Panel (c) shows the distribution of mapping quality (MAPQ) of all the reads in a representative sample. MAPQ is defined as -10×log_10_ *Pr(mapping position is wrong)*, rounded to the nearest integer. As shown on the x-axis in (c), MAPQ ranges between 0 and 60 in this sample, with 60 indicating the best mapping. Y-axis in (c) is the number of reads in this sample. Panel (d) shows the distribution of MAPQ of all the reads in a sample and the reads mapped to *CDC27* exon regions. Y-axis in (d) is the proportion of reads in each MAPQ range (x-axis).

**Figure 7:**
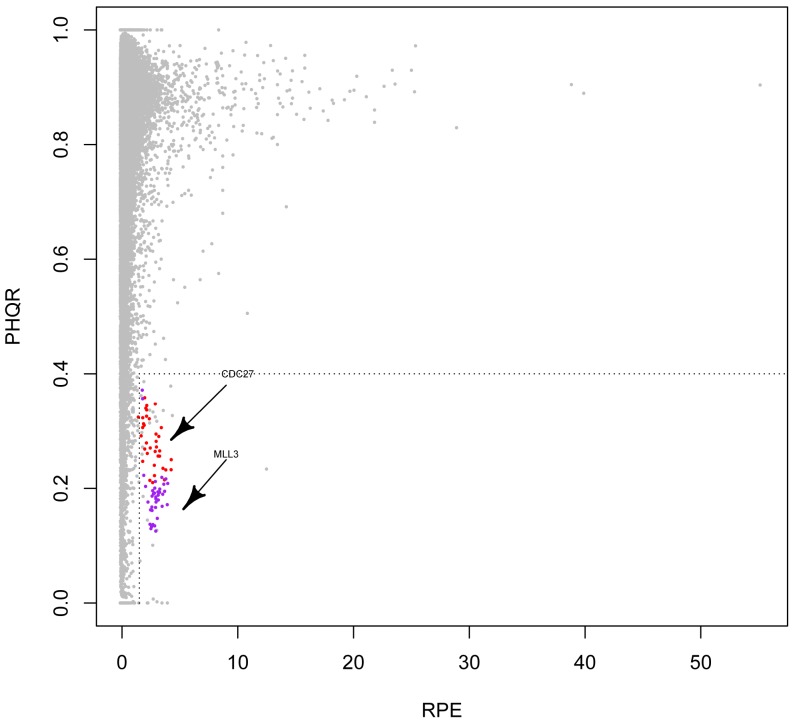
Detection of spurious genes. *RPE:* the number of Reads Per Exon after adjusting the length of the exon and the overall sequencing depth per sample. *P_HQR_*: the Proportion of High-Quality Reads for each exon. Each point represents an exon. The grey points represent all the exons in one sample. The red points indicate the distribution of the 13^th^ exon of the gene *CDC27* in all 36 samples, and purple points indicate the distribution of the 42^nd^ exon of the gene *MLL3* in all 36 samples, both of which are representative spurious genes and failed to be validated by experiments. The vertical dash line is set *RPE*  = 1.5 and the horizontal dash line is set *P_HQR_*  = 0.4.

**Table 2:** Spurious genes having mutations detected in >30 samples.

CCDS ID	Gene symbol	Exon	# samples
CCDS11509.1	*CDC27*	13^th^	36
CCDS12749.1	*CGB*	3^rd^	36
CCDS12752.1	*CGB5*	1^st^	36
CCDS41378.1	*NBPF11*	19^th^	36
CCDS43407.1	*FAM153C*	4^th^	36
CCDS5931.1	*MLL3*	42^nd^	36
CCDS34703.1	*STAG3*	33^rd^	34
CCDS5590.1	*POMZP3*	1^st^	34
CCDS10638.1	*EIF3C*	8^th^	32
CCDS30836.1	*NBPF14*	22^nd^	31

CCDS: Consensus coding sequence. Exon: the specific exon in which the variants are detected.

To explore systematically the existence of spurious genes, we carefully examined several known spurious genes/mutations (e.g., *CDC27*) and found that they tend to have two features: (1) high coverage around the variation site, which could be partially explained by incorrect assignment of reads (see above), and (2) low quality of local alignment. Figure 6 shows an example of good alignment that has most of the bases matched perfectly to the reference genome except at the SNV site (Figure 6a), and an example of bad alignment that has many mismatched bases within each read (Figure 6b, the local alignment around two exons of *CDC27*). Additionally, a long segment of the sequences around the target regions is normally involved. Hence, the bad alignment will exist across several bases rather than only the targeted site. Therefore, we recommend describing the “local alignment environment” of a variant locus rather than only considering the alignment at the locus. In this work, we considered each exon as an analysis unit.

For each exon, we proposed two parameters to quantitatively measure these features in order to facilitate a manual check of high-risk spurious loci/genes. First, to assess the coverage of an exon, we derived a parameter *RPE* to denote the normalized number of Reads Per Exon: 
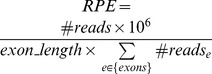
. In this formula, the absolute number of reads for an exon is normalized (1) by its length (*exon_length*) so that different exons with different lengths are comparable to each other, and (2) by the total number of reads mapped to exon regions (
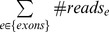
) for each sample so that the same exon in different samples can be compared. Secondly, we define a parameter *P_HQR_* to compute the Proportion of High-Quality Reads (HQRs) for each exon. In the alignment results by BWA, each read is assigned a mapping quality (MAPQ), defined as -10×log_10_ *Pr(mapping position is wrong)* and rounded to the nearest integer [22]. The MAPQ values range between 0 and 60, with higher values indicating high mapping quality (Figure 6c). We used the MAPQ information to indicate if a read has high-quality if its MAPQ was higher than or equal to 40, and the proportion of such high-quality reads for each exon was then computed as *P_HQR_*. The cutoff value of 40 was selected based on the overall distribution of mapping quality (Figure 6c). Of note, a pre-filtering step on MAPQ is expected to be executed on the initial alignment file (BAM/SAM) before SNV/indel calling, e.g., the command line “*SAMtools view -q 1*” is suggested to remove reads mapped to multiple positions by the “*-q*” option [23]. However, the value used in this step (*-q*) is typically not as high as 40, because this setting could be too strict and, in turn, remove numerous reads. In summary, in the alignment files used for SNV/indel calling, the MAPQ values vary widely, and *P_HQR_* could be used to indicate the overall alignment quality.

Figure 7 displays the distribution of *P_HQR_* versus *RPE* in our samples. In the figure, each point represents an exon and grey nodes indicate the cohort of all exons. We specifically examined the distribution of the gene *CDC27* (CCDS11509.1) and its 19 exons in all 36 samples. Here, we also included the matched control samples because the spurious gene phenomenon is a systematic artifact and is expected to occur in any samples regardless of disease status. As shown in Figure 7, one exon of *CDC27* was observed to depart from the major distribution (red points), and this phenomenon occurred in all the 36 samples. In the plot panel (Figure 7), this exon is located in the right bottom area where the coverage is high (x-axis), and the proportion of high-quality reads is low (y-axis), which is consistent with the two features we have expected (see above). We manually set this region as *RPE*>1.5 and *P_HQR_*<0.4 and then collected all the exons that were located in this area. After ranking these exons according to their number of occurrence samples, we identified a total of 10 genes observed in more than 30 samples (83% of 36 samples) (Table 2). Again, these cutoff values are arbitrary. Researchers may apply a more stringent cutoff to require occurrence in >90% of samples or refine the area in the plot by setting *RPE* and *P_HQR_*. However, one may need to be cautious of the possibility false positives regarding the exons we listed in Table 2. Manual examination of local alignment should be performed before experimental validation to save efforts and resource.

The gene/exon lists, either by Ju et al. [28] or by our work, and cutoff values for spurious genes may vary depending on different runs and platforms. However, the main features of high coverage and low proportion of high-quality reads are typical for these genes. While the genes in Table 2 display the need for investigators to take extra caution, we suggest they check for the local alignment or draw similar figures in their specific NGS projects. Manual examination of the local alignment environment could remove most of these genes effectively. This examination is performed after SNV/indel calling and before experimental validation.

In conclusion, we systematically examined the major factors that could potentially improve validation rates in next-generation sequencing data and summarized four parameters aiming to provide general guidelines. These parameters are (1) both quality and read depth are important factors in variant detection; (2) realignment and recalibration help improving variant calling; (3) there are allele and strand difference between the positions that have been successfully validated and those that have failed in validation; and (4) manual check could help filter spurious genes. These points provide useful and timely guidelines in the selection of software/pipelines for calling SNVs/indels and in the follow up selection of variants for validation. A high validation rate not only reduces the cost and labors in experimental validation of NGS data, but also avoids reporting false discoveries in literature or public databases. Although we identified these points primarily based on exome data, they could also readily be applicable to WGS data.

## Materials and Methods

A total of 18 lung tumor:normal pairs were captured using the Agilent SureSelect 38 M kit and sequenced on an Illumina HiSeq 2000 platform. On average, 48 Mb paired-end reads were generated per sample with an average sequencing depth of 63× on targeted regions. This study was approved by the Institutional Review Board of the Fudan University Shanghai Cancer Center, Shanghai, China. All participants gave written informed consent. Details of the sequencing strategy and description of the datasets are provided elsewhere (manuscript in preparation).

The overall pipeline is shown in Figure 1. Briefly, all three resultant variant sets were based on the same mapping results initially generated using BWA [20] to map reads of each sample to the human reference genome (hg18). Duplicate reads and reads with a Phred-based quality score <15 were removed from subsequent analyses. Cleaned alignment result files in the BAM format were then prepared for variant calling. The NGS data analyses were conducted in a high performance computing cluster comprising 3700 processor cores and having a theoretical peak performance of 12 TeraFLOPS available at the Vanderbilt Advance Computing Center for Research and Education (ACCRE, http://www.accre.vanderbilt.edu/).

For the tier one variant set, we called SNVs/indels using the SAMtools:*mpileup* function and filtered the resultant variations using the *varFilter* function provided by the vcfutils.pl script in SAMtools using “*-d3 -D10000*” option, i.e., requiring 3 or more but no more than 10,000 read depth for each putative variant. For the tier two variant set, we performed base quality score recalibration and local realignment around known indels based on the initial alignment results, followed by SNV/indel detection in the same way we did for the tier one set using the SAMtools:*mpileup* function and filtering. For the tier three variant set, we called SNVs/indels using the initial alignment files as we used for tier one set, but we used the SAMtools:*pileup* function. A filtering step using “$3 =  = “*”&&$6> = 50) || ($3! = “*”&&$6> = 20” was applied on the resultant file by *varFilter* implemented in the samtools.pl script [23]. The detailed functional commands are available upon request.

## Supporting Information

Figure S1 **Distribution of Pearson correlation coefficient of the QUAL values by SAMtools:** ***pileup*** **and SAMtools:** ***mpileup*** **in one representative lung cancer sample.** Each node represents one putative SNV or indel called by both functions. The red line is y = x.
(PDF)
