# Supplementary figures and images for: Consensus Rules in Variant Detection from Next-Generation Sequencing Data
Source: PLoS One. 2012 Jun 8;7(6):e38470. doi: 10.1371/journal.pone.0038470 (PMC3371040; doi:10.1371/journal.pone.0038470)

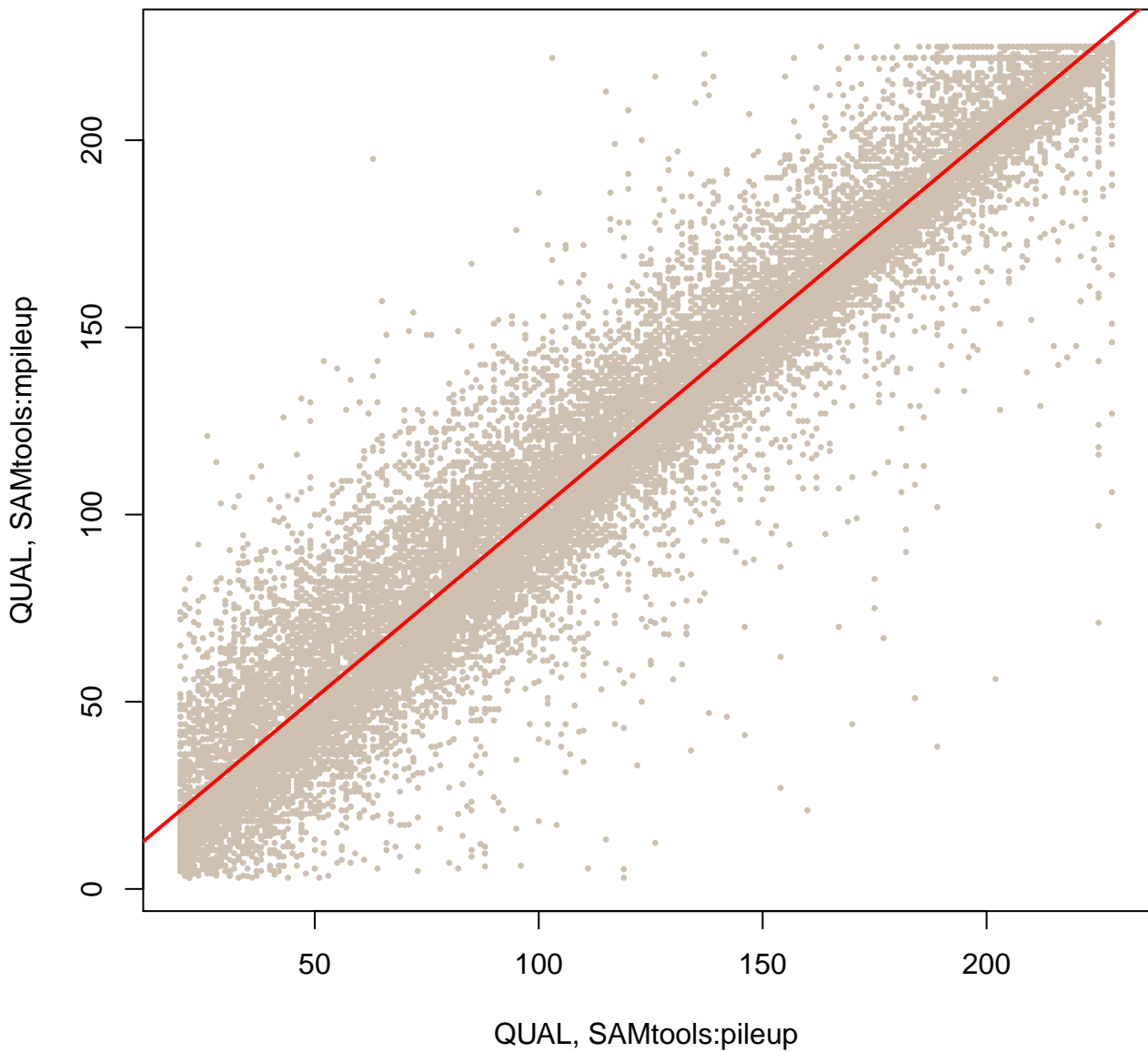

Supplement: Figure S1 — Distribution of Pearson correlation coefficient of the QUAL values by SAMtools: pileup and SAMtools: mpileup in one representative lung cancer sample. Each node represents one putative SNV or indel called by both functions. The red line is y = x. (PDF) [file pone.0038470.s001.pdf]
